# Corrigendum to Cross‐sectional area reference values for peripheral nerve ultrasound in adults: A systematic review and meta‐analysis—Part I: Upper extremity nerves

**DOI:** 10.1111/ene.16373

**Published:** 2024-06-11

Fisse AL, Katsanos AH, Gold R, Pitarokoili K, Krogias C. Cross‐sectional area reference values for peripheral nerve ultrasound in adults: a systematic review and meta‐analysis—Part I: Upper extremity nerves. Eur J Neurol. 2021 May;28(5):1684–1691. doi:10.1111/ene.14759.

In the abstract, the results and table 1 the value for the calculated mean cross‐sectional area (CSA) of the ulnar nerve in the upper arm was incorrect (6.6 mm^2^). The correct value for the CSA of the ulnar nerve in the upper arm is 5.6 mm^2^.

We apologize for this error.

